# Percutaneous epiphysiodesis transphyseal screw versus tension-band plating as hemiepiphysiodesis in treating coronal angular knee deformities: a systematic review and meta-analysis of comparative studies

**DOI:** 10.1186/s12891-025-08540-z

**Published:** 2025-04-11

**Authors:** Ahmed O. Sabry, Mohamed K. A. Genedy, Salma Abouelwafa, Ahmed A. Khalil, Omar Mady, Noureldin Mostafa, Rana Ali Ahmed Elsayed, Hassan Elbarbary, Mohamed Hegazy, Amr Abdelgawad

**Affiliations:** 1 Orthopaedic Department, Faculty of Medicine, Cairo University, El Saray Street Manial - El Manial, Cairo, 11956 Egypt; 2 Orthopaedic Research Network, Faculty of Medicine, Cairo University, Cairo, Egypt; 3 Maimonides Medical Center, Brooklyn, NY USA

**Keywords:** Angular knee deformities, Genu varum, Genu valgum, Hemiepiphysiodesis, Percutaneous epiphysiodesis transphyseal screw, Tension-band plating, Systematic review, Meta-Analysis

## Abstract

**Background:**

Angular knee deformities such as genu varum and genu valgum are common in children and can impact their functional mobility and quality of life. Although surgical interventions like guided growth plates or tension-band plates (TBP) and percutaneous epiphysiodesis transphyseal screws (PETS) are commonly used, comparative analyses of their efficacy and safety are limited. This study aims to evaluate the correction rates and safety profiles of TBP and PETS in treating pediatric coronal angular knee deformities.

**Methods:**

A comprehensive literature search was conducted in Scopus, Web of Science, and PubMed until November 2024. Only comparative clinical studies comparing PETS and TBP in pediatric patients with coronal knee deformities were included.

**Results:**

A total of five studies encompassing 473 physes were included. Their methodological quality was assessed using the MINORS criteria, with scores ranging from 18 to 19, indicating a low risk of bias. PETS demonstrated significantly higher correction rates compared to TBP, with an overall pooled mean difference in angular correction of 0.17°/month (*p* < 0.0003). In the femoral subgroup analysis (LDFA), the mean difference correction rate was 0.21°/month in favor of PETS (*p* = 0.01). Additionally, the PETS group achieved a statistically significant mechanical axis deviation mean difference correction rate of 1.02 mm/month (*p* = 0.006). Complication rates were relatively lower with PETS across all included studies.

**Conclusion:**

PETS achieves faster angular and mechanical axis deviation correction rates compared to TBP, highlighting its efficiency in treating pediatric coronal angular knee deformities. Additionally, PETS demonstrates relatively fewer complications, reinforcing its position as a more effective and cost-efficient option for guided growth in children.

**Clinical trial number:**

Not applicable.

**Level of evidence:**

II.

## Introduction

Angular knee deformities, including genu varum (bowlegs) and genu valgum (knock-knees), are relatively common, with a prevalence roughly estimated to range at around 0.2% [1]. While many cases of angular knee deformities resolve naturally or with conservative management, some require surgical intervention [2, 3].

Traditionally, treating angular knee deformities was done by corrective osteotomy and casting. Osteotomy, which involves surgically realigning the bone to correct malalignment, has proven effective but is highly invasive, carrying significant risks such as infection, delayed healing, over or undercorrection and potential occurrence of compartment syndrome or neurovascular injury due to the acute correction performed [4].

This has led to the development of instrumented guided growth techniques, which utilize reversible procedures to temporarily slow growth on the overgrown side of the physis, allowing the angular deformity to gradually correct as the child continues to grow. The concept of influencing bone growth by manipulating the physis dates back to the 19th century, with early observations by surgeons like Hueter, Volkmann, and Delpech, who noted the effects of pressure on physeal growth [5]. This foundational understanding eventually led to the development of open epiphysiodesis techniques to achieve permanent growth arrest for corrective purposes, as first described by Phemister in 1933 [6, 7]. Later, Haas introduced instrumentation that enabled reversible growth inhibition by removing the device after achieving the desired correction [8].

Since then, several hemiepiphysiodesis implants have been developed. Staples, first introduced by Blount, were among the earliest implants used for guided growth [9]; however, they were prone to high rates of mechanical failure. As a rigid implant, staples exerted fixed pressure on the physis, leading to the growth plate shifting away from the tips of the staples. This displacement often resulted in staple expulsion, limiting their long-term effectiveness [10, 11]. This challenge was addressed with the development of the tension-band plate (TBP), a system consisting of two non-locked screws connected by a small plate, this design introduced flexibility into the construct, allowing it to function as a dynamic tension band rather than a rigid fixation device [12]. Unlike staples, TBP does not apply immediate and direct compressive force; instead, it places the fulcrum outside the bone. This alters the distribution of compressive forces on the physis, offering a more physiologically compatible mechanism for guided growth correction and reducing complications associated with rigid implants [13, 14].

Another option for guided growth is the percutaneous epiphysiodesis transphyseal screw (PETS) introduced in 1998 by Métaizeau [15], which has promising results reported in the literature, including faster correction rates and minimally invasive application [16–18].

Despite their widespread use, direct comparisons of TBP and PETS in the literature remain scarce. While previous meta-analyses have focused on these implants as a form of epiphysiodesis for leg length discrepancies [19], this meta-analysis is the first to comprehensively evaluate their angular correction rates, overall correction efficacy, and associated complication rates as a form of hemiepiphysiodesis for angular knee deformities.

## Methodology

### Protocol registration

We registered our systematic review and meta-analysis on PROSPERO (CRD42024612039) and followed the PRISMA guidelines as well as the Cochrane Handbook for Interventions [20, 21]. Clinical trial number: not applicable.

### Data sources & search strategy

In November 2024, a comprehensive search across three databases (PubMed, Web of Science Core Collection, and Scopus) to identify studies comparing PETS and TBP in coronal angular knee deformities was conducted. We used the following search string: (Genu OR Knee OR leg) AND (valgum OR varum OR valga OR varus OR knocking OR bow OR deformity OR deformities) AND (((8 OR eight OR tensions) AND (plate OR band)) OR (PETS OR percutaneous epiphysiodesis OR transphyseal screw*)). In addition, we searched specific journals relevant to the field and cross-referenced the relevant text.

### Eligibility criteria

We included comparative studies with two treatment groups directly assessing correction rates of guided growth procedures using either TBP or PETS in skeletally immature children with coronal plane knee deformities. Follow-up was required until appropriate correction of the deformity was achieved. Studies limited to single treatment arms, case reports, or secondary analyses were excluded.

### Study selection

After duplicate removal by Sciwheel (Digital Science, London, United Kingdom), articles were imported into the Rayyan web tool (Rayyan Systems, Inc., Cambridge, MA). Two authors initially screened titles and abstracts independently. Afterward, full-text articles were collected for a secondary blind screening conducted within Rayyan.

### Data extraction

The authors collected quantitative and qualitative data for each outcome from the included studies. The extracted data were recorded in two standardized spreadsheets. The first sheet was for study and demographic characteristics such as study ID, study design, age, and sex. The second sheet was dedicated to primary and secondary outcomes, including correction rates for the mechanical axis deviation (MAD), medial proximal tibial angle (MPTA), lateral distal femoral angle (LDFA), and complication rates. Web-PlotDigitizer (Technology from Sage, Thousand Oaks, CA) was used to extract data from various plots when needed.

### Quality assessment

Two independent authors conducted a quality assessment using the Methodological Index for Non-Randomized Studies (MINORS) criteria [22]. The included studies were evaluated across 12 methodological domains, assessing the reporting of study aims, endpoint assessment, follow-up, statistical analysis, and other areas. Each domain receives a score from 0 to 2, where 0 is not reported, 1 is inadequately reported, and 2 is adequately reported.

### Statistical analysis

RevMan 5.4 (RevMan web, London, UK) was utilized to pool the mean differences (MD) for MAD and angular correction rates, standardized to millimeters per month (mm/month) and degrees per month (°/month), respectively, across three subgroups: MPTA, LDFA, and a non-specific correction rate. For one study, where standard deviations were not reported, Wan’s formula was applied to facilitate meta-analysis [23]. Heterogeneity was assessed using the Chi² and the I² statistic. A *p*-value < 0.05 was considered statistically significant. Low, moderate, and high heterogeneity were defined as I² < 25%, 25–75%, and > 75%, respectively. To account for heterogeneity, a random effect model was employed to pool MD, and sensitivity analysis was conducted to assess the validity of the results.

## Results

### Search results and study selection

Our comprehensive search across three databases initially identified 2,538 records. After removing 1,167 duplicates, 1,371 records remained for screening. Of these, 1,364 were excluded due to the wrong study design, implant type, or population not involving hemiepiphysiodesis. Seven full-text articles were evaluated for eligibility. Two studies were excluded: one utilized screws combined with nonabsorbable sutures as a tension band rather than a TBP, and the other focused solely on epiphysiodesis, which did not meet the inclusion criteria.

**Fig. 1:**
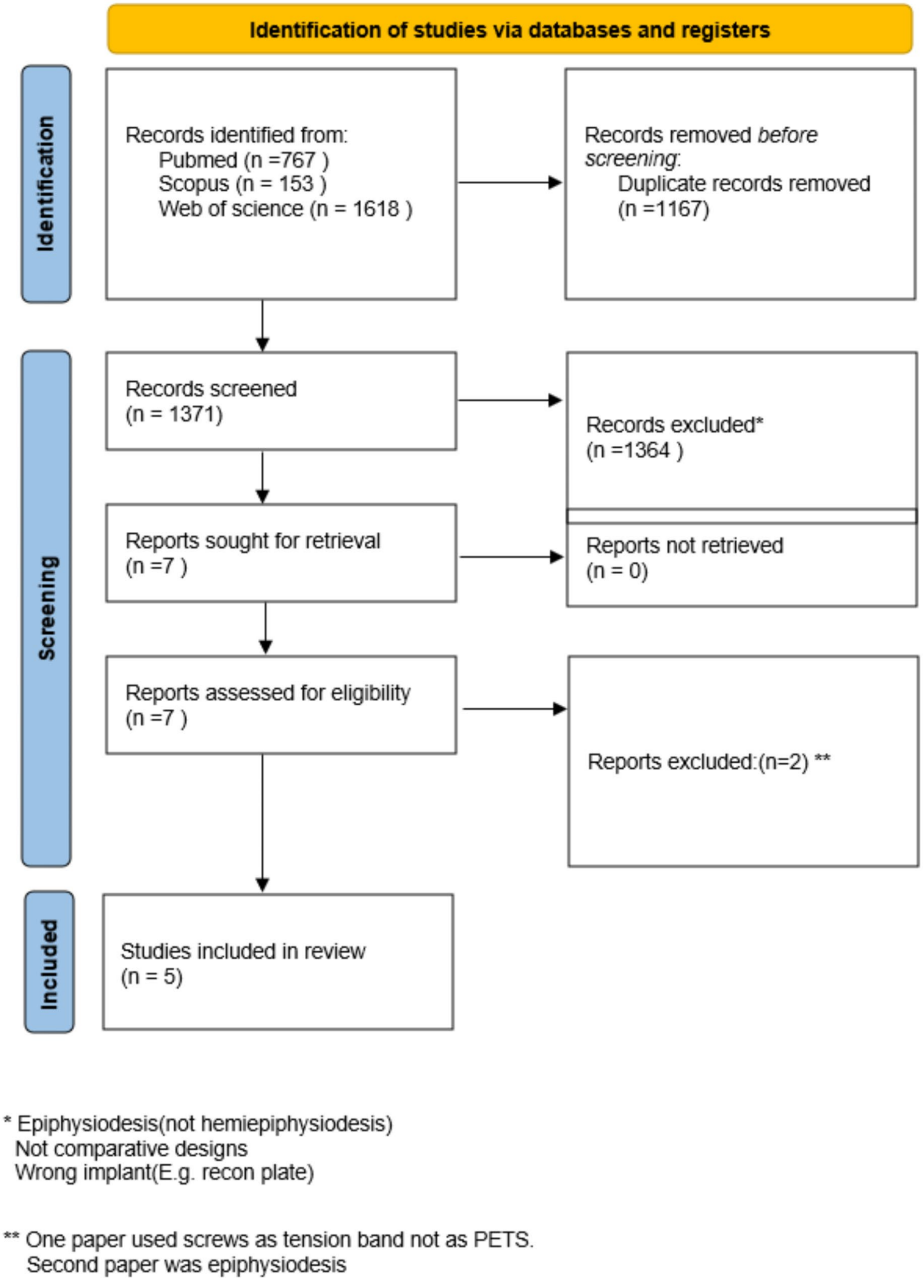
PRISMA flow chart

Ultimately, five studies were included in the review [24–28]. The study selection process is outlined in the PRISMA flow diagram (Fig. 1).

### Characteristics of included studies

Table 1 summarizes the characteristics of the five included studies [25–28], which involved a total of 228 patients (129 males, 99 females) and ranged from 30 to 57 patients per study, with an average mean age of 12 years and ranges of 7 to 15 years across studies, with the mean body mass index varying from 21 to 23.6 kg/m².

**Table 1:** Summary of studies’ characteristics

Author, Country, Year, and Journal	Study Design	Male: Female: Total Number of Patients	Limbs		Physes			Mean Body Mass Index (kg/m^2)	Mean/median Age (years)	
			Valgus	Varus	T: F: Total	TBP (T: F)	PETS (T: F)		TBP	PETS
Heckel, Germany, (2023). Journal of Limb Lengthening & Reconstruction.	Retrospective Cohort Study	25: 19: 44 patients (49 angular corrections as 5 cases needed repeat surgeries)	41 patients	8 patients	61: 72: 133	28:42	33:30	NR	11.3 (3.6–15.8) years	13.1 (10.9–15.8) years
McGinley, United States. (2023) The Journal of Bone and Joint Surgery.	Retrospective Cohort Study	35: 27: 62 patients	4 patients	58 patients	0: 62: 62	0:31	0:31	NR	12.9 ± 1 years	12.8 ± 1.2 years
Park, South Korea (2022). BMC Musculoskeletal Disorders.	Retrospective Cohort Study	12: 18: 30 patients	30 patients	0	23: 50: 73	10:27	13:23	23.6 ± 4.4 kg/m^2^	11.2 ± 1.7 years	11.2 ± 1.7 years
Shapiro, Israel (2022). Archives of Orthopedic and Trauma Surgery.	Retrospective Cohort Study	25: 10: 35 patients	27 patients	8 patients	NR: NR: 55 physes	Total 23 physes	Total 32 physes	NR	13 (7–15) years	13 (7–15) years
Park, South Korea (2016). Journal Of Pediatric Orthopedics.	Retrospective Cohort Study	32: 25: 57 patients	57 patients	0	60: 90: 150 physes	20:40	34:56	21 ± 3.8 kg/m^2	11.7 (8.2–14.4)	12.1 (9.4–14.4)

The number of included total physes across studies ranged from 55 to 150, with a total of 473 physes included in this review. Studies included both distal femoral and proximal tibial physes, with the exception of McGinley et al. who exclusively used femoral physes [28]. These studies were conducted in diverse settings, including Germany [27], the United States [28], South Korea [25, 26], and Israel [24], between 2016 and 2023. All studies employed a retrospective cohort design and examined TBP and PETS interventions for angular knee deformities.

### Quality assessment

The studies’ total scores ranged from 18 to 19 across 12 domains of MINORS criteria [22]. All included studies [25–28] adequately reported five domains (aims, endpoints, follow-up periods, statistical analyses and control and contemporary groups). However, all studies reported six domains inadequately (consecutive patients, unbiased assessment of endpoints, prospective sample size calculation, and data collection domains), which is inherently due to the studies being retrospective. For the baseline equivalence domain, all studies except Heckel et al. [27] demonstrated adequate baseline equivalence. McGinley et al. [28] employed a matched study design, while the remaining studies reported no significant differences between groups at baseline (Table 2).

**Table 2:** Summary of MINORS quality assessment

Study Id	A clearly stated aim	Inclusion of consecutive patients	Prospective collection of data	Endpoints appropriate to the aim of the study	Unbiased assessment of the study endpoint	Follow-up period appropriate to the aim of the study	Loss to follow-up less than 5%	Prospective calculation of the study size	An adequate control group	Contemporary groups	Baseline equivalence of groups	Adequate statistical analyses	Total score
Heckel et al. 2023	2	1	1	2	1	2	1	1	2	2	1	2	18
McGinley et al. 2023	2	1	1	2	1	2	1	1	2	2	2	2	19
Park et al. 2022	2	1	1	2	1	2	1	1	2	2	2	2	19
Shapiro et al. 2022	2	1	1	2	1	2	1	1	2	2	2	2	19
Park et al. 2016	2	1	1	2	1	2	1	1	2	2	2	2	19

### Meta-analysis results

The meta-analysis assessed the angular correction rates of two treatments, PETS and TBP, for coronal angular knee deformities across five studies involving 473 physes [24–28].

#### Angular correction rates

**Fig. 2:**
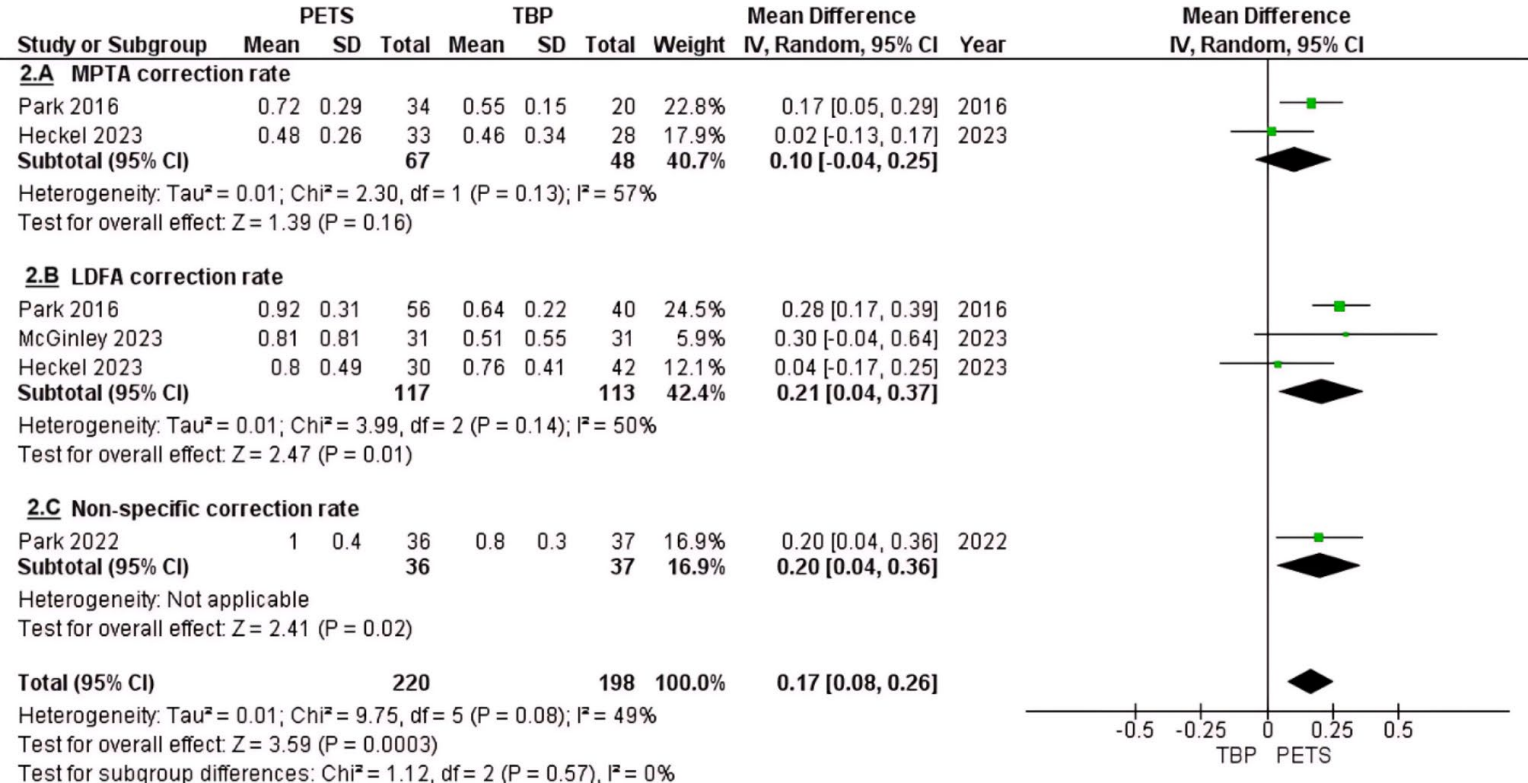
Angular correction rate forest plot. (**A**) MPTA correction rate subgroup. (**B**) LDFA correction rate subgroup. (**C**) non-specific correction rate subgroup

The angular correction rates were analyzed across three subgroups: MPTA, LDFA, and a non-specific subgroup. The MPTA subgroup included two studies, Park et al.(2016) and Heckel et al., with a total of 115 tibial physes. The mean difference (MD) in favor of PETS was 0.1°/month (95% CI: -0.004 to 0.25; *p* = 0.16) (Fig. 2A). While the results suggested a trend favoring PETS, statistical significance was not achieved.

The lateral distal femoral angle (LDFA) subgroup included three studies, Park et al.(2016), Heckel et al., and McGinley et al., with 230 femoral physes. The MD was 0.21°/month in favor of PETS, demonstrating statistical significance (95% CI: 0.04 to 0.37; *p* = 0.01) (Fig. 2B).

Park et al. (2022) did not differentiate MPTA from LDFA when reporting angular corrections and was thus placed in a separate non-specific subgroup. The MD was 0.20°/month in favor of PETS, with statistical significance (95% CI: 0.04 to 0.36; *p* = 0.02) (Fig. 2C).

An overall pooled MD was calculated for all the angular correction subgroups, and was found to be in favor of PETS at 0.17°/month (*p* < 0.0003) (Fig. 2).

**Fig. 3:**
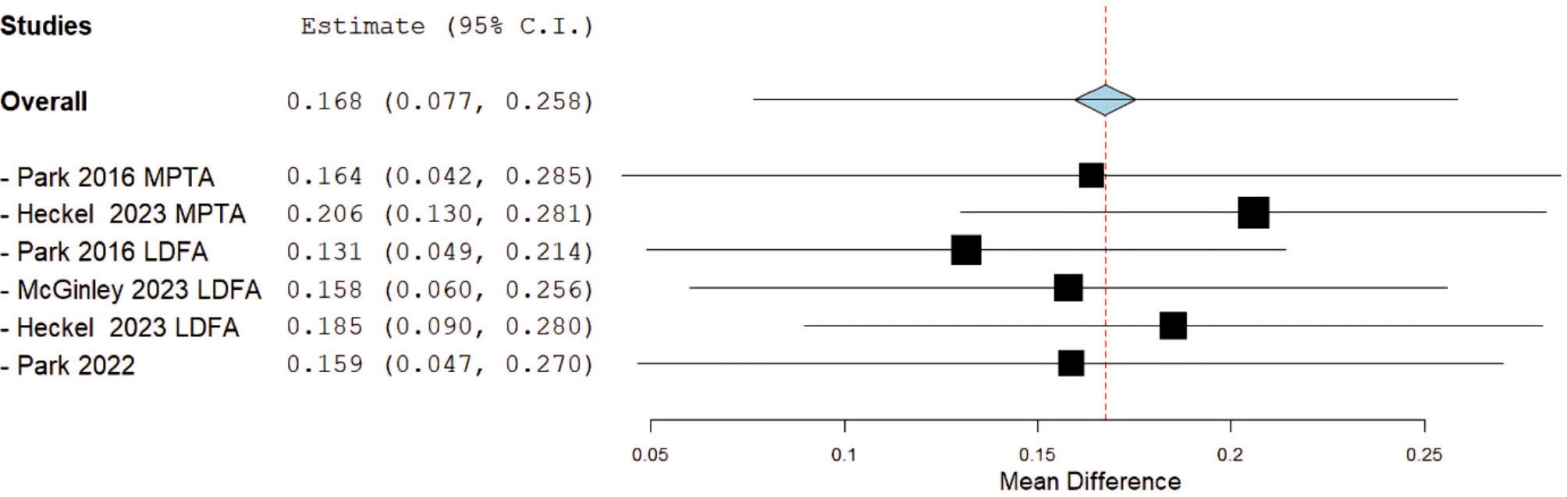
Angular correction rate sensitivity analysis plot

Heterogeneity wasn’t statically significant across the overall analysis and subgroups (I²= 48–56%; *P* > 0.05), and sensitivity analysis showed the robustness of the results with the overall effect remaining in favor of PETS with a significant difference regardless of which study is removed by applying the leave-one-out (Fig. 3).

#### Mechanical axis deviation correction rates

**Fig. 4:**
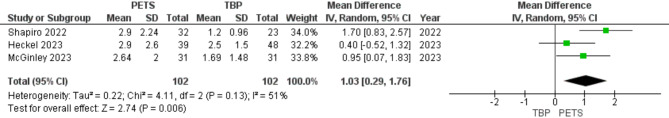
MAD correction rate forest plot

The second outcome measure analyzed was the mechanical axis deviation (MAD) correction rate. This analysis included three studies: Shapiro et al., Heckel et al., and McGinley et al. The results demonstrated a significant MD of 1.02 mm/month in favor of PETS (95% CI: 0.29 to 1.76; *p* = 0.006) with non-statically significant heterogeneity (Fig. 4).

### Complications

Complications associated with guided growth were reported in four studies. However, the level of detail varied significantly between the studies, preventing us from pooling the results. Park et al. (2016) did not include an assessment of complications in their findings [25], while McGinley et al. noted fewer complications in the PETS group but did not specify the types or frequencies of these complications [28]. As for Heckel et al., a need for 5 reoperations was reported of which 4 were initially treated with TBP [27]. The PETS group consistently demonstrated a lower complication rate across all studies, with the exception of overcorrection, which was specifically reported by Park et al. (2022) [26]. TBP exhibited a complication rate of up to three to six times higher than that of PETS in some studies [24, 26], with the rebound phenomenon emerging as the most commonly reported issue [26]. There were isolated cases of implant failure reported by Shapiro et al. [24]., as well incisional complications, predominantly reported in the TBP group, and were attributed to the larger incision required for this technique [24] (Table 3).

**Table 3:** Summary of complications

Study Id	TBP group complications	PETS group complications
Heckel et al. 2023	4/25 cases (16%) additional operation due to an unspecified indication.	1/19 cases (5%) additional operation due to an unspecified indication.
McGinley et al. 2023	?/31 physes (?%) Higher rebound rates	?/31 physes (?%) Fewer complications.
Park et al. 2022	18/37 physes (49%) rebound phenomenon 1/37 physes (3%) overcorrection	4/36 physes (11%) rebound phenomenon 11/36 physes (31%) overcorrection
Shapiro et al. 2022	4/23 physes (18%) Incisional complications: Surgical Site Infection Hypertrophic Scarring Incisional Pain 1/23 physes (4.5%) plate failure	1/32 physes (3%) Incisional complications: Surgical Site Infection 1/32 physes (3%) screw loosening
Park et al. 2016	NR	NR

## Discussion

To the best of our knowledge, the efficacy of guided growth devices has been compared statistically by a meta-analysis in leg length discrepancy only [19]. Our meta-analysis is the first to present a statistical comparison of the efficacy of PETS and TBP in correcting angular knee deformities and assess the safety of each implant across comparative studies.

**Fig. 5:**
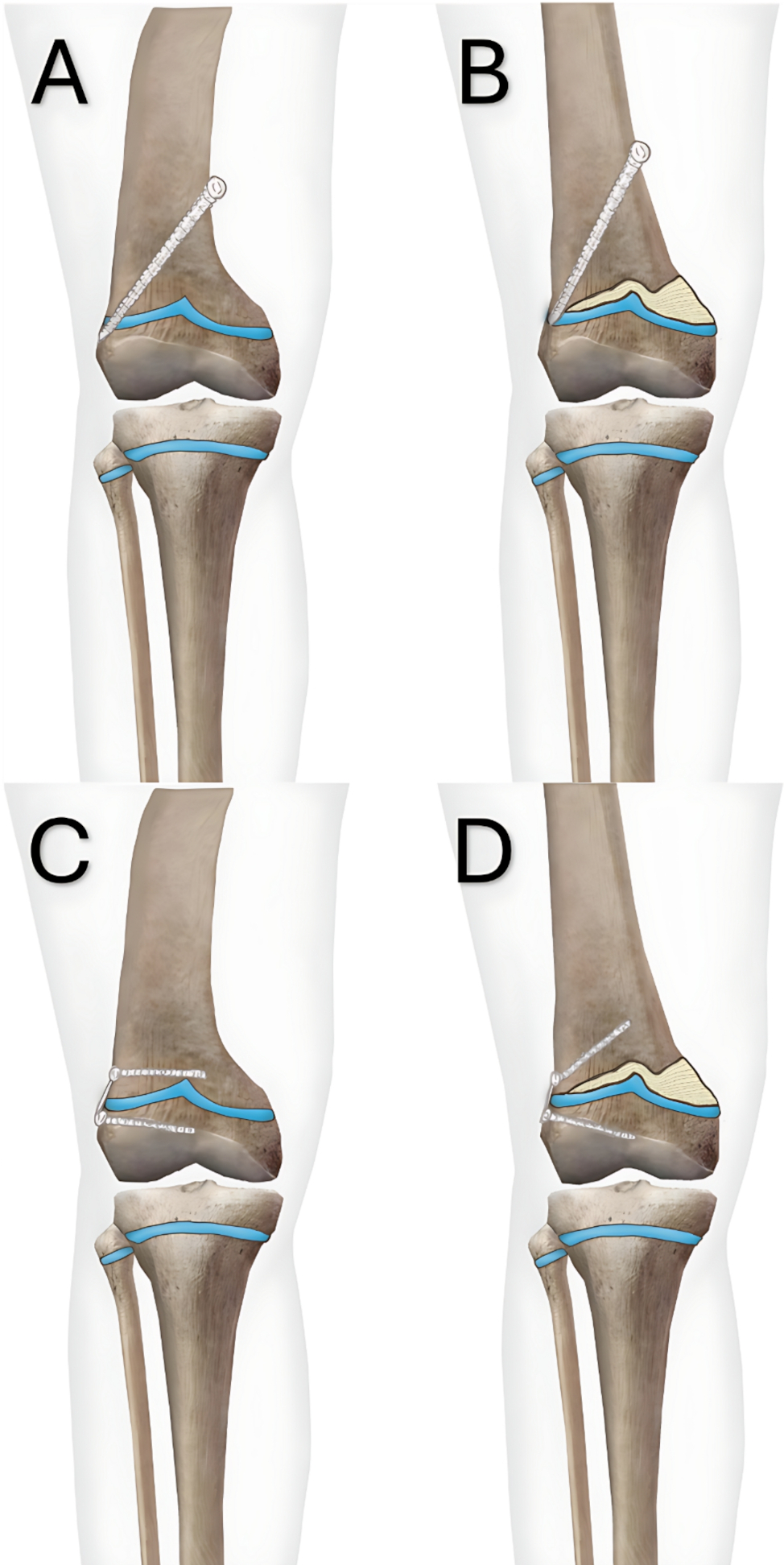
Mechanisms of PETS and TBP: (**A**) PETS is inserted obliquely, with threads crossing the physis to limit growth, halting it immediately. (**B**) As the concave side lengthens, the screw head anchors and may pull threads out of the physis upon correction. (**C**) TBP is fixed with screws placed parallel to the physis. (**D**) Growth tension pushes the flexible screws outward, causing divergence and halting growth

This study demonstrates that PETS achieves faster angular correction rates and MAD correction rates compared to TBP, which was consistently observed in the overall correction. The accelerated correction with PETS can be attributed to its direct mechanical influence on immediate physeal growth, facilitated by its transphyseal configuration [29, 30]. In contrast, the lower correction rate associated with TBP likely arises from delayed compression of the growth plates. In the TBP mechanism, tension builds gradually on the screws as growth progresses, eventually creating a hinge-like effect at the physis perimeter until growth stops, taking time until its full effect takes place [25, 28] (Fig. 5).

Complications and the need for additional operations were more frequent in the TBP group than in the PETS group across all studies, see (Table 3). TBP showed a higher rebound phenomenon rate, likely due to the release of tension on the physis after device removal, which allowed for a transient increase in growth on the controlled side [31]. Risk factors such as high angular deformity and younger patient age, may increase susceptibility to rebound [32, 33]. Some authors advocate for routinely applying a 5-degree overcorrection as a preventive measure against rebound when using TBP [34–36]. However, Leveille et al. suggest reserving overcorrection for patients identified as having a high risk of rebound, cautioning that unnecessary overcorrection in low-risk patients may result in the development of a new deformity [37].

Additionally, TBP had higher incisional-related complications, including issues related to larger incision size, which predisposed patients to surgical site infections, hypertrophic scarring, and incisional pain, as noted by Shapiro et al., with a complication rate of 18%, compared to 3% in the PETS group, most likely due to larger incision size, and dissection through muscle and fascia [24].

Concerns have been raised regarding the potential risk of growth arrest due to PETS perforating the physes. However, no instances of physeal arrest were reported in the included studies following screw removal. Multiple studies have confirmed that normal physeal growth resumes after PETS screws are removed [17, 38]. However, Park et al. (2022) observed higher rates of overcorrection of the deformity in the PETS group after the removal of the screw [26]. This could be explained by the presence of a physeal bar that is present in the physis where the screw had passed through before. The incidents were temporary, as reported by Park et al. (2022), and none of the cases had permanent growth arrest on the affected side [26]. To minimize the risk of potential permanent physeal injury, careful surgical technique is still essential during PETS placement. Drilling should stop just short of the physis, and the screw should pass smoothly through without excessive force. Precision from the outset minimizes the risk of creating a larger physeal bar, ensuring a reversible and safer correction process.

Factors such as three-point bending stress caused by the gap between the metaphyseal hole and the bone have been proposed as contributors to TBP loosening and screw breakage [39, 40]. However, in our included studies, only an isolated case was reported by Shapiro et al. [24]. However, other studies have documented significantly higher rates of screw breakage and failure, particularly in cases involving Blount’s disease [39, 41].

When considering the use of TBP for angular deformity correction, it is important to distinguish between idiopathic and pathological physes. TBP is associated with low complication rates in cases of mild angular deformities with idiopathic causes. However, its performance significantly declines in pathological physes, where failure rates are markedly higher—reaching up to 45% in patients with pseudoachondroplasia and 36.8% in those with Blount disease [39, 42–44], with the metaphyseal screw breaking. In such cases, the use of solid screws or transitioning to PETS is recommended to reduce the risk of failure and improve outcomes.

Economically, PETS appears to be a more cost-effective option than TBP constructs, as it incurs lower overall expenses due to its cheaper price and reduced need for additional procedures for complications [27, 28].

### Limitations

Due to scarce literature, we included five studies, all of which were retrospective studies with small sample sizes, limiting our results’ generalizability. Furthermore, inconsistencies in outcome reporting prevented the inclusion of complication data in the meta-analysis.

We identified a potential partial overlap between the patient populations of Park et al. (2022) and Park et al. (2016). However, this does not affect the integrity of our results for several reasons. First, no data from both studies were included in the same forest plot, eliminating the possibility of skewing the meta-analysis results. Second, Park et al. (2016) did not report complication rates, ensuring that safety outcomes remain unaffected. Lastly, even after performing a leave-one-out sensitivity analysis—where we sequentially removed each study and recalculated the pooled correction rate, the overall angular correction rate statistical significance in favor of PETS remained unchanged, confirming the robustness of our findings (Fig. 3).

### Future research recommendations

Future research should focus on prospective, matched studies to validate our findings on the efficacy of PETS and TBP. To facilitate further statistical analyses, standardized reporting is crucial. Key details, including the number of patients, treated limbs, and physes, as well as the specific types of physes and treatment groups, should be documented for each outcome. Additionally, a comprehensive assessment of complications is vital, as the safety profile is a defining factor that sets hemiepiphysiodesis apart from other treatment modalities.

## Conclusion

PETS achieves faster correction rates and hence a shorter period of treatment and may be a more cost-effective option than TBP, as it requires cheaper implants and is associated with lower reoperation rates, and possibly relatively safer than TBP for treating angular knee deformities.

## Data Availability

The data that support the findings of this study are available within the study.
